# Translation, Cross-Cultural Adaptation, and Validation of the Malay Version of the Protection Motivation Theory Scale Questionnaire for Pap Smear Screening

**DOI:** 10.3390/ijerph19116858

**Published:** 2022-06-03

**Authors:** Rodziah Romli, Emma Mirza Wati Mohamad, Rahana Abd Rahman, Kah Teik Chew, Syahnaz Mohd Hashim, Azmawati Mohammed Nawi

**Affiliations:** 1 Department of Community Health, Faculty of Medicine, Universiti Kebangsaan Malaysia, Cheras, Kuala Lumpur 56000, Malaysia; rodziah@moh.gov.my; 2 Institut Latihan Kementerian Kesihatan Malaysia Alor Setar, Ministry of Health, Alor Setar 05250, Malaysia; 3 Centre for Research in Media and Communication (MENTION), Faculty of Social Sciences and Humanities, Universiti Kebangsaan Malaysia, Bangi 43600, Malaysia; emmamohamad@ukm.edu.my; 4 Department of Obstetrics and Gynecology, Faculty of Medicine, Universiti Kebangsaan Malaysia, Cheras, Kuala Lumpur 56000, Malaysia; drrahana@ppukm.ukm.edu.my (R.A.R.); drchewkt@ukm.edu.my (K.T.C.); 5 Department of Family Medicine, Faculty of Medicine, Universiti Kebangsaan Malaysia, Cheras, Kuala Lumpur 56000, Malaysia; syahnaz@ppukm.ukm.edu.my

**Keywords:** protection motivation theory, cross-cultural adaptation, cervical cancer screening, Malay translation

## Abstract

Pap smear screening can detect cervical cancer early, but is underutilized. Motivational factors play a role in ensuring that women undergo Pap smear screening. This study was conducted to validate the adapted instrument, which was based on the protection motivation theory (PMT), into the Malay language to evaluate the motivational factors for Pap smear screening among women. The original 26-item PMT scale was developed based on seven constructs of the PMT framework. The adaptation involved translation by bilingual experts (*n* = 4), followed by synthesis (*n* = 6). Subsequently, we performed content validation (content validation index, CVI) among the health experts (*n* = 5) and face validation (face validation index, FVI) among women (*n* = 11). Reliability testing for internal consistency was determined via the confirmatory factor analysis (CFA) of women aged between 21 and 65 years (*n* = 150). One item was deleted based on the expert consensus, leaving a total of 25 items after the adaptation. The validation yielded a good CVI and FVI. Prior to CFA reliability testing, one item was deleted due to very low factor loading. The CFA indicated a good fit for 24 items. The factor loading (range: 0.45–0.98), average variance extracted (range: 0.44–0.90), and composite reliability (range: 0.69–0.97) indicated that the convergent validity for each construct was acceptable, except for the perceived vulnerability. However, the perceived vulnerability construct was accepted based on expert verification. We confirmed that the translation, cross-cultural, adaptation, and validation of the Malay-version PMT scale were valid and reliable. The scale contains 24 items that represent the seven constructs of the PMT framework.

## 1. Introduction

Cervical cancer (CC) remains a reproductive health burden and a global health issue. The World Health Organization (WHO) presented a global strategy toward eliminating CC from public health concerns. The vision included a threshold age-standardized rate (ASR) of 4.0 per 100,000 women, with 70% of women screened before the age of 35 years and rescreened by 45 years [1]. Almost 90% of death due to CC occurs in developing countries and low- to middle-income countries (LMICs) as a consequence of the less successful implementation of screening methods [2,3,4]. In Malaysia, 1 in 144 women is diagnosed with CC at an ASR of 6.2 per 100,000 women [5]. The coverage of women undergoing Pap smear screening in Malaysia in 2014–2018 (i.e., 5 years) remained below 40% (23–26%) [6]. The availability of free screening programs at health clinics throughout Malaysia has failed to encourage women to undergo Pap smear screening.

In light of this, motivational factors may play a role in improving the CC screening rate among Malaysian women. The protection motivation theory (PMT) explains how an individual is motivated toward self-protection against health threats [7,8,9]. The PMT framework has been proven to motivate women toward increasing the CC screening rates [10,11,12]. The framework assumes that protection motivation (the individual’s intention to perform a behavior) results from two appraisal processes: The positive function, which includes perceived vulnerability, perceived severity, self-efficacy, and response efficacy; and the negative function of the rewards associated with fear (threat appraisal) and the response costs (coping appraisal) of the adaptive behavior.

The factors that influence knowledge, attitude, awareness, and barriers to CC and Pap smear screening in Malaysia have been identified [13,14,15]. They include less knowledge on the causes and clinical manifestations of CC [13,14] and insufficient information from health care professionals regarding CC and Pap smear screening [13,14]. The factors associated with uptake of Pap smear screening include taking hormonal contraception, higher knowledge and attitude scores [14], and the use of text reminders as educational material [15]. However, there has been no investigation into the motivation and personal potential variables. Hence, we assessed how motivation can influence screening behavior by translating the available PMT scale [16] into the Malay language and performed cross-cultural adaptation.

The original PMT scale was developed by Laleh Hassani in 2014 for Iranian women to measure the intention for the first Pap smear screening [16]. The instrument is a theory-based framework that is suitable for developing educational intervention material on Pap smear screening [12]. This new Malay-version PMT scale can be used as an instrument to assess women’s motivation toward Pap smear screening and guide the development of an intervention module in the future.

## 2. Methods

Overview: In order to produce a Malay-version PMT, this study performed the adaptation process including the translation process and synthesis. We further validated the cross-cultural adaptation items via content validation by expert and face validation by women. The validated item was tested for reliability testing using confirmatory factor analysis (CFA) before the finalized Malay-version PMT was formed.

The original PMT scale consists of 26 items encompassing seven constructs scored on a 5-point Likert scale from 0 (completely disagree) to 5 (completely agree). The score is calculated based on individual constructs and can range from 2 to 30. The seven constructs are: perceived vulnerability, perceived severity, response efficacy, self-efficacy, fear (threat appraisal), response cost (coping appraisal), and behavioral intention (protection motivation) toward Pap smear screening. The exploratory factor analysis (EFA) performed by the original author affirmed that the developed PMT scale had a suitable structure [Kaiser–Meyer–Olkin = 0.82; Bartlett’s test of sphericity = 3911.78 (degree of freedom [df] = 406, *p* < 0.0001)] and the seven constructs jointly accounted for 72.76% of the variance [16]. The EFA is appropriate for scale development, in order to identify the nature of the constructs whereas the CFA is preferred for measurement models or to establish the validity of a set of measures [17,18]. The nature of constructs was discovered by the original author, thus this study proceeded with CFA to test the adaptation set of constructs for internal consistency. We used IBM SPSS Amos version 24 for the statistical analysis.

Permission to use and translate the PMT scale into Malay was obtained from the corresponding author. Creating the Malay-version PMT scale began with adaptation, which included forward- and back-translation and synthesis. This was followed by validation, which consisted of content validity, face validity, and reliability testing.

### 2.1. Adaptation

The original PMT scale was adapted based on the cross-cultural adaptation by referring to the guidelines of Beaton and Bombardier [19] and consist of translation, synthesis, back-translation, expert committee review, and pre-testing for an understanding of the items.

### 2.2. Translation

The forward- and back-translation involved four bilingual expert translators with the following qualifications: Masters in Education and Professional Studies, Master of Education in TESL (Teaching English as a Second Language), or Bachelor of Education [Teaching English to Speakers of Other Languages (TESOL)]. The forward-translation (from English to Malay) was conducted by two expert bilingual translators and each translator produced a report of the translation. This was followed by a back-translation (Malay to English) by another two expert bilingual translators, and two translation reports were produced independently.

### 2.3. Synthesis

The four translation reports were combined into one document after a thorough discussion between the translators and two public health physicians to address any gaps or differences between the respective reports. The process included the decision for the best Malay word to be used that was easy to understand, and suitable phrases for adaptation into the Malaysian culture.

### 2.4. Validation

#### 2.4.1. Content Validity

Content validation began with a panel of five experts competent in both languages (consisting of a gynecologist, public health physician, family medicine specialist, public health nurse, and health educator information officer) who reviewed the final synthesized report of the translation by comparing each item in both languages (i.e., Malay and English versions). The experts were also asked to check the content of the PMT scale to ensure that the items were culturally appropriate to the Malaysian population.

After completing the cultural adaptation, the experts assessed the content validity index (CVI) to calculate the item-level content validity index (I-CVI) and the scale-level content validity index-average (SCVI-average). The CVI is an index of inter-rater agreement among experts. The experts were asked to evaluate whether the items of the PMT scale were relevant, clear, and essential by rating each item on a 4-point Likert scale (1 = not relevant, 2 = somewhat relevant, 3 = quite relevant, 4 = very relevant). Items with a rating of 1 and 2 were considered invalid and items with a rating of 3 and 4 were considered valid. The CVI was computed by calculating the scale average. An I-CVI of 0.78 and SCVI-average of ≥0.90 indicated that the item had good content validity [20].

#### 2.4.2. Face Validity

Eleven women aged 21–65 years and of different ethnicities, residence locations (urban or rural), education levels, and occupational statuses were purposively selected for pre-testing on face validation for understanding of the items. The face validation testing was aimed at assessing the clarity and comprehensibility of the translated items. The women were required to award scores from 1 (item not clear and not understandable) to 4 (item very clear and understandable) based on the clarity and comprehensibility of the translated items in the PMT scale. Scores of 3 and 4 were recategorized as 1 (clear and understandable) and scores of 1 and 2 were recategorized as 0 (not clear and understandable). The face validity index (FVI) was computed by calculating the scale average [20].

### 2.5. Reliability Testing

Reliability testing for internal consistency via confirmatory factor analysis (CFA) was conducted online through Google Forms in multiple WhatsApp groups among 150 women aged 21–65 years throughout Malaysia. The sample size should be greater than the number of items (i.e., N > p), with the recommended N:p ratios ranging from 5 with a minimum N > 100 [21,22]. As the PMT scale contained a total of 26 items, the sample size of 150 was adequate and exceeded the minimum sample requirement of 130.

As previous studies have determined the factors and items of the PMT scale [16], we only conducted a confirmatory study of the translated PMT scale among the 150 women. The study aimed at confirming whether the seven constructs involving the 26 items fit the measurement model well. To achieve good psychometric characteristics, high standardized factor loadings (>0.40) are preferred [23,24]. Higher factor loading values indicate higher levels of reliability. Values between 0.60 and 0.70 are considered to be “acceptable in exploratory research” and values of 0.70–0.90 range from “satisfactory” to “good” [24].

Additionally, we examined the average variance extracted (AVE) regarding the grand mean value of the squared loadings of the indicators associated with the construct. An AVE of ≥0.50 indicates that the construct explains ≥50% percent of the variance of the indicators that comprise the construct [24]. The composite reliability (CR) represents the aggregation of the indicators under a latent variable with a value that should be >0.6 [24,25]. If the AVE is <0.5 but the CR is >0.6, the convergent validity of the construct remains adequate [26]. To confirm the fitness of the CFA measurement model, the fitness of the measurement model for the construct validity assessment was required to yield the following values: comparative fit index (CFI) and goodness fit index (GFI) > 0.90, root mean square error of approximation (RMSEA) < 0.08, and chi-square (ChiSq/df) < 5 [26,27].

## 3. Results

### 3.1. Cross-Cultural Adaptation

Prior to the forward and back-translation and synthesis, several changes were made to adapt the PMT scale to the Malaysian culture. The term “husband” in item PS3 was changed to “partner”, as unmarried women who are sexually active should also undergo CC screening. Item SE1 was deleted as the statement was more toward the behavioral intention rather than self-efficacy. Furthermore, the word “periodically” was added to item SE7 to emphasize the need for repeat screening. Items PM1 and PM2 were repositioned because the “intend” statement should precede the “plan” statement (Table 1).

### 3.2. Content Validity and Face Validity Index

The inter-expert CVI was 0.8–1 with an SCVI-average of 0.90. The inter-women FVI was 0.8–1 with an SFVI-average of 0.95. The results were considered to indicate good CVI and FVI [20]. Table 1 shows the cross-cultural adaptation, synthesis changes, CVI, and FVI of the Malay version of the PMT scale.

### 3.3. Descriptive Analysis for Reliability Testing

Table 2 lists the characteristic profiles of the women involved in the reliability testing. The mean age was 39.5 years (SD 9.69), the majority were of Malay ethnicity (78%), married (78.7%), had higher education levels (74.7%), were employed (70.7%), had a personal income of <MYR4851 (68%), and were urban residents (70.7%). The majority were sexually active (76.7%), had heard of CC screening (96.7%), and had not undergone CC screening in the last 3 years (70%).

Normal data distribution was confirmed using basic descriptive analyses examining the pattern and shape of the sample distribution on the PMT scale. Table 3 lists the analyses results. The mean score for each construct was as follows: perceived vulnerability, 11.56 (SD 2.18); perceived severity, 15.82 (SD 2.78); fear (threat appraisal), 8.30 (SD 3.41); response costs (coping appraisal), 6.03 (SD 2.54); response efficacy, 17.69 (SD 2.81); self-efficacy, 22.85 (SD 5.53); and protection motivation, 12.72 (SD 3.04). The skewness and kurtosis values were within the acceptable range of normal distribution of the dataset score from −2 to +2 [28].

### 3.4. Confirmatory Factor Analysis

The CFA was performed on 25 items of the PMT scale after one item had been deleted during the synthesis changes of cross-cultural adaptation. Factor loading for all items was >0.4 and ranged from 0.45 (PS4) to 0.98 (PM3); the exceptions were for items PV1 (0.07) and PV3 (0.37). Item PV1 was eliminated from the measurement model due to the very low factor loading. Item PV3 was maintained as its factor loading was close to 0.4 if rounded and because the item was required for complementing the perceived vulnerability construct, as suggested by the expert verification (Table 4).

The Cronbach’s alpha, AVE, and CR for all constructs was accepted based on their values of >0.6, 0.4, and 0.6 respectively. Thus, the convergent validity was accepted for perceived severity, fear (threat appraisal), response cost (coping appraisal), response efficacy, self-efficacy, and protection motivation. The exception was perceived vulnerability, which yielded an alpha = 0.35, AVE = 0.24, and CR = 0.37, which were less than the expected value. Due to the need to retain the construct, verification was obtained from the same expert in the cross-cultural adaptation. Table 4 shows the overall CFA results.

**Figure 1:**
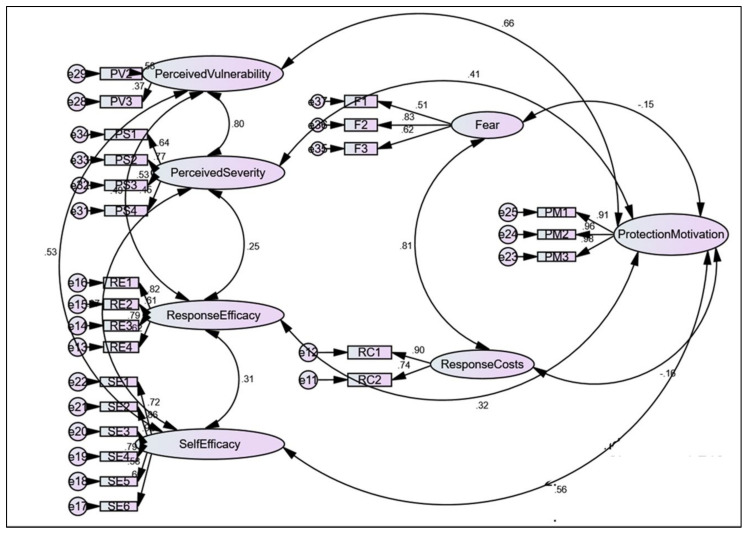
The finalized overview of the confirmatory factor analysis. The circle shape represents the unobserved variables, the rectangle shape represents the observed variables of PMT items (PV = perceived vulnerability, PS = perceived severity, RE = response efficacy, SE = self-efficacy, F = fear, RC = response costs, PM = protection motivation) and the oval shape represents the seven PMT constructs. The double headed arrow shows the correlation between the PMT construct.

After deleting item PV1, the fitness of the measurement model for construct validity assessment was fit based on the ChiSq/df = 1.746; RMSEA = 0.071; GFI = 0.814, and CFI = 0.906. Table 5 summarizes the difference before and after PV1 deletion according to the description of the fitness indices. Figure 1 presents the finalized overview of the CFA among the seven constructs and 24 items of the Malay-version PMT scale.

## 4. Discussion

The translation, cross-cultural adaptation, and validation of the Malay-version PMT scale yielded satisfactory reliability and validity results for the 24 items when tested among women throughout Malaysia. One item of the self-efficacy construct (*I will take have the Pap*) was deleted during cross-cultural adaptation. Ideally, the adaptation of the questionnaire into a different culture can affect the actual meaning of the words. The original PMT scale was in Farsi [16] and the item *“I will take have the Pap”* was acceptable as a self-efficacy construct based on the local context. However, the content validity expert in the present study stated that the item was more appropriate as a protection motivation (intention) construct when translated into Malay. The translation process may encounter difficulty when two different languages have nonequivalent words and may lead to different meanings [29]. In addition, the cross-cultural adaptation process not only focuses on the translation, but also on an appropriate cultural adaptation that is suitable for a new setting [19]. The justification to the adaptation process is the ability to place the host culture [30] as well as achieve a balance between the original source and the new version of the adaptation [19]. Thus, the decision to delete the item was appropriate.

One item of the perceived vulnerability construct (*I do not have any problems in my reproduction organ, so it is impossible to have cervical cancer*) was deleted during CFA reliability validation due to the very low factor loading (0.07). After deleting the item, the fitness of measurement model for the construct validity assessment was fit (ChiSq/df = 1.746, RMSEA = 0.071, GFI = 0.814, CFI = 0.906). The item was identified as a reverse code item during the translation. Thus, this might have affected the women’s understanding when selecting the appropriate scale for the item and resulted in the very low factor loading of the CFA. The other item of perceived vulnerability construct (*Among my relatives, no one has cervical cancer and neither do I*) was accepted, even though the factor loading was 0.37, which was slightly less than the acceptable value (>0.4). This item needed to be retained to achieve the perceived vulnerability construct. Prior to the expert’s content validation, the translation of the item into Malay was meaningful and the item was necessary to evaluate the perceived vulnerability construct. The decision on whether to delete or retain an item was subject to the content validity expert, wherein weaker loadings could be retained [24].

Based on our results, the Malay-version PMT scale was acceptable for Malaysian women in different demographic settings. Our selection criteria were women aged 21–65 years who were eligible for CC screening without limiting for other factors. Our aim was to test the clarify and reliability of the adapted validated instrument among Malaysian women. Thus, the reliability testing was conducted electronically via Google Forms in multiple WhatsApp groups throughout Malaysia. With this method, we were able to eliminate geographic barriers and capture a variety of demographic settings. The majority of our respondents were Malay, with higher education levels, and from urban areas. Nevertheless, the characteristics of the other ethnicities also contributed to the finalized CFA model. Mohamad Marzuki [29], who used the same approach via WhatsApp groups, also captured the same respondent characteristics that we did.

Malaysia has a multi-ethnic population that uses Malay as the national language. To identify the clarity of the content, we selected Chinese and Indian women as representatives of other ethnic groups during the face validation. The I-FVI (0.8–1.0) and SFVI-average (0.95) proved the clarity of the items among a multi-ethnic group. Furthermore, the involvement of 22% non-Malay respondents (Chinese, Indian, natives of Sabah, and natives of Sarawak) in the reliability testing also contributed to the acceptance-finalized model of the CFA. This respondent heterogeneity may be extended to the findings and can be generalized to all Malaysian women.

Although our findings confirmed the finalized fitness model of the Malay-version PMT scale, the use of Google Forms during the reliability testing might have affected our findings. The respondents answered the online questionnaire without guidance and this could have affected their understanding of the items. This limitation could also have affected the deleted item.

## 5. Conclusions

We confirmed that the translated, cross-culturally adapted, and validated Malay-version PMT scale as a valid and reliable tool to be used in the Malaysian population. This 24-item instrument represents the seven constructs based on the PMT framework: perceived vulnerability, perceived severity, fear (threat appraisal), response cost (coping appraisal), response efficacy, self-efficacy, and protection motivation (intention). The Malay-version PMT scale is valid for assessing women’s motivation toward CC screening. Further research is needed to explore the promotion of lifelong screening among women and developing tailored interventions based on the PMT scale.

## Figures and Tables

**Table 1:** The cross-cultural adaptation, content validity, and face validity index of the Malay version of the PMT scale.

PMT Construct	Item	English Version	Synthesis Changes	I-CVI of Experts (*n* = 5)	I-FVI of Women (*n* = 11)
Perceived vulnerability	PV1	I do not have any problems in my reproduction organ, so it is impossible to have cervical cancer.		1	1
	PV2	I worry about having cervical cancer.		1	1
	PV3	Among my relatives, no one has cervical cancer and neither do I.		0.8	1
Perceived severity	PS1	Cervical cancer imposes high expenditure on me and my family		0.8	1
	PS2	If I have cervical cancer, my life will change.		1	1
	PS3	Cervical cancer limits me from having sex with my husband. (partner) *	“husband” to “partner”	0.8	1
	PS4	If I have cervical cancer, I will die in five years.		1	1
Fear (Threat Appraisal)	F1	I fear that the Pap test confirms my cancer.		1	1
	F2	I am afraid of the examination pain.		1	1
	F3	I fear that Pap smear confirms a problem in my reproduction organ.		1	1
Response costs (Coping Appraisal)	RC1	The Pap test is not pleasant for me.		1	1
	RC2	I am ashamed to have the Pap test.		1	1
Response efficacy	RE1	The Pap test is effective in preventing cervical cancer.		1	1
	RE2	The Pap test helps with early diagnosis of the disease.		1	1
	RE3	The Pap test prevents the progress of cervical cancer.		1	0.8
	RE4	Early diagnosis using the Pap test saves the patient’s life.		1	1
Perceived self–efficacy	SE1	I will take have the Pap. *	item drop out	-	-
	SE2	I have the Pap test even if I do not have enough money.		0.8	1
	SE3	I have the Pap test even if it is painful.		1	1
	SE4	I have the Pap test despite being shameful.		1	1
	SE5	I have the Pap test even if I am busy.		1	1
	SE6	I have the Pap test even if my relatives refrain from it.		0.8	1
	SE7	I would repeat the Pap test in the coming years (periodically) *, even if its result is negative.	add on “periodically”	1	1
Protection Motivation (Intention)	PM1	I plan to have the Pap test. *	swap to PM2	0.8	1
	PM2	I intend to have the Pap test. *	swap to PM1	0.8	1
	PM3	I want to have the Pap test.		0.8	1
			SCVI-average	0.90	-
			SFVI-average	-	0.95

* I-CVI = item-level content validity index; I-FVI = item-level face validity index; SCVI-average = scale-level content validity index; SFVI-average = scale-level face validity index.

**Table 2:** The demographic profile of women for reliability testing.

Variables		*n*	%	Mean ± SD
Age	≤40 years old	97	64.7	39.5 (9.69)
	≥41 years old	53	35.3	
Ethnic group	Malay	117	78.0	
	Non-Malay	33	22.0	
Marital status	Unmarried	26	17.3	
	Married	118	78.7	
	Divorced/Separated/Widower	6	4.0	
Educational level	≤Secondary education	38	25.3	
	Higher education (Certificate/Diploma/Degree)	112	74.7	
Occupational status	Employee	106	70.7	
	Self-employed	12	8.0	
	Not employed	32	21.3	
Estimated personal income	≤B40 * (below MYR4850)	102	68.0	
	>B40 (MYR4851 and above)	48	32.0	
Location of residence	Urban	106	70.7	
	Rural	44	29.3	
Sexually active for at least six months	Yes	115	76.7	
	No	35	23.3	
Having heard of cervical cancer screening	Yes	145	96.7	
	No	5	3.3	
Have had cervical screening in the last 3 years	Yes	45	30.0	
	No	105	70.0	

* B40 = Bottom 40% (Lower-income group with household income is below MYR 4850 per month).

**Table 3:** The description of the PMT scale construct.

		Total PV	Total PS	Total F	Total RC	Total RE	Total SE	Total PM
N	Valid	150	150	150	150	150	150	150
Missing		0	0	0	0	0	0	0
Mean		11.56	15.82	8.30	6.03	17.69	22.85	12.72
Std. Deviation		2.178	2.783	3.409	2.537	2.807	5.528	3.037
Skewness		−0.850	−0.773	−0.095	−0.083	−1.100	−0.475	−1.450
Std. Error of Skewness		0.198	0.198	0.198	0.198	0.198	0.198	0.198
Kurtosis		1.911	0.400	−0.807	−1.046	0.117	−0.222	1.669
Std. Error of Kurtosis		0.394	0.394	0.394	0.394	0.394	0.394	0.394

Total PV = total score of perceived vulnerability; Total PS = total score of perceived severity; Total F = total score of fear; Total RC = total score of response cost; Total RE = total score of response efficacy; Total SE = total score of self-efficacy; Total PM = total score of protection motivation.

**Table 4:** The confirmation factor analysis (CFA).

PMT Construct	Item	Factor Loading	Alpha	AVE	CR	Convergent Validity
Perceived Vulnerability	PV1 ^r^	0.07				
	PV2	0.58	0.35 *	0.24 *	0.37 *	Expert verification
	PV3	0.37 *				
Perceived Severity	PS1	0.64	0.67	0.44	0.69	Accepted
	PS2	0.77			0.69	
	PS3	0.53				
	PS4	0.45				
Response Efficacy	RE1	0.82	0.78	0.51	0.81	Accepted
	RE2	0.61				
	RE3	0.79				
	RE4	0.62				
Self-Efficacy	SE1	0.72	0.89	0.58	0.89	Accepted
	SE2	0.86				
	SE3	0.91				
	SE4	0.79				
	SE5	0.58				
	SE6	0.66				
Fear (Threat appraisal)	F1	0.51	0.73	0.44	0.70	Accepted
	F2	0.83				
	F3	0.62				
Response Costs (Coping appraisal)	RC1	0.90	0.80	0.68	0.81	Accepted
	RC2	0.74				
Protection Motivation (Intention)	PM1	0.91	0.97	0.90	0.97	Accepted
	PM2	0.96				
	PM3	0.98				

* expert verification; ^r^ = reverse coded items; Alpha = Cronbach’s alpha; AVE = average variance extracted; CR = composite reliability.

**Table 5:** The fitness of measurement model for the construct validity assessment.

Fitness Indexes	Value before Item Deleted	Value after * PV1 Deleted	Acceptable Value	Description
ChiSq/df	1.752	1.746	<5	Model vs. Saturated
RMSEA	0.071	0.071	<0.08	Root mean Squared Error of Approximation
GFI	0.805	0.814	>0.9	Comparative Fit Index
CFI	0.897	0.906	>0.9	Tucker-Lewis Index

* PV1 = Item 1 in perceived vulnerability construct.

## Data Availability

The data presented in this study are available in this article.
